# Cold Agglutinin-Mediated Hemolysis as an Extrapulmonary Manifestation of Mycoplasma pneumoniae Infection: A Case Report

**DOI:** 10.7759/cureus.102165

**Published:** 2026-01-23

**Authors:** Sara Isabel Vasconcelos, Beatriz Vitó Madureira, Catarina Branco, Sofia Perestrelo Lima, Rita Soares Costa

**Affiliations:** 1 Internal Medicine, Unidade Local de Saúde de Entre Douro e Vouga, Santa Maria da Feira, PRT

**Keywords:** cold agglutinin hemolysis, community-acquired pneumonia, extrapulmonary manifestations, hematologic complications, mycoplasma pneumoniae

## Abstract

*Mycoplasma pneumoniae* (*M. pneumoniae*) is a frequent cause of community-acquired pneumonia (CAP) in young adults and may present with extrapulmonary manifestations, including hematologic complications such as cold agglutinin-mediated hemolysis. We report the case of a previously healthy 20-year-old Caucasian man who presented with a four-day history of fever, asthenia, anorexia, and dry cough. Imaging revealed extensive left-sided pulmonary consolidation, and empirical therapy for CAP was initiated. During hospitalization, new-onset anemia with erythrocyte agglutination following blood warming raised suspicion of immune-mediated hemolysis, later supported by positive cold agglutinins and serologic confirmation of *M. pneumoniae* infection. The patient showed progressive clinical and analytical improvement with targeted azithromycin therapy. He remained asymptomatic and hematologically stable at the three-month follow-up. This case reinforces the importance of recognizing significant hematologic manifestations that may accompany or even precede respiratory involvement in *M. pneumoniae* infections. Early identification of cold agglutinin-associated hemolysis can expedite diagnostic evaluation and guide appropriate therapy, contributing to favorable outcomes.

## Introduction

*Mycoplasma pneumoniae* (*M. pneumoniae*) is one of the most common atypical pathogens associated with community-acquired pneumonia (CAP), particularly among adolescents and young adults [1]. The clinical presentation is usually insidious, beginning with headache, asthenia, and fever, and may progress to persistent cough and respiratory failure. In addition to pulmonary involvement, approximately 25% of cases present with extrapulmonary manifestations affecting multiple systems, including skin (Stevens-Johnson syndrome, erythema multiforme), gastrointestinal tract (abdominal pain, diarrhea), nervous system (encephalitis, meningoencephalitis), and cardiovascular (myocarditis, pericarditis, pericardial effusion) and hematologic systems [2,3].

Among the hematologic complications, cold agglutinin-induced hemolysis is particularly notable. This phenomenon results from cross-reactivity between *M. pneumoniae* antigens and the erythrocyte membrane I antigen, leading to the production of antibodies that can trigger hemolysis at low temperatures [4,5]. While cold agglutinins are often observed in the setting of *M. pneumoniae* infection, clinically overt cold agglutinin autoimmune hemolytic anemia seems to be a rare phenomenon, with reported presentations spanning from mild and transient anemia to severe, life-threatening hemolysis. When present, it is typically supported by laboratory findings such as a positive direct antiglobulin test, elevated lactate dehydrogenase, reduced haptoglobin, reticulocytosis, and indirect hyperbilirubinemia [4,6].

Extrapulmonary manifestations may precede, accompany, or even dominate the clinical presentation, potentially complicating diagnosis and delaying appropriate treatment. Early recognition is therefore essential [7].

This case is presented to highlight the need to consider significant hematologic manifestations in the context of *M. pneumoniae* CAP, even in previously healthy young adults, and to emphasize the importance of a high index of clinical suspicion [4].

## Case presentation

A 20-year-old Caucasian man with no relevant past medical history presented to the emergency department with a four-day history of fever, asthenia, anorexia, and dry cough. Upon admission, he was afebrile and hemodynamically stable. Pulmonary auscultation revealed preserved vesicular breath sounds, and no adventitious sounds were heard. Arterial blood gas analysis demonstrated mild hypoxemic respiratory failure. Laboratory studies showed elevated inflammatory markers (Table 1).

**Table 1:** Laboratory parameters at admission, during hospitalization, and follow-up Trends in hematologic, inflammatory, hepatic, and renal parameters from admission through hospital day 3, discharge, and outpatient re-evaluation. Persistent erythrocyte agglutination after sample warming to 37 °C was observed until discharge, with resolution documented at follow-up.

Parameter	Reference range	Admission	Hospital day 3	Discharge	Follow-up
Hemoglobin	12-15 g/dL	13.6	12.9	14.0	16.7
Mean corpuscular volume	80.0-100.0 fL	89.8	91.5	90.3	94.1
Erythrocyte series	-	Agglutination at room temperature that resolved after warming to 37°C	Agglutination at room temperature that resolved after warming to 37°C	Agglutination at room temperature that resolved after warming to 37°C	Normal
Leukocytes	4.00-11.00 ×10⁹/L	8.3	8.7	7.9	6.2
Neutrophils	1.80-8.00 ×10⁹/L	6.49	6.32	4.60	2.86
Lymphocytes	1.00-4.50 ×10⁹/L	1.05	1.86	2.67	2.53
Monocytes	0.20-1.00 ×10⁹/L	0.65	0.42	0.40	0.47
Eosinophils	0.10-1.00 ×10⁹/L	0.03	0.00	0.00	0.26
Basophils	0.02-1.00 ×10⁹/L	0.04	0.05	0.10	0.06
Platelets	150-400 ×10⁹/L	320	361	369	275
Lactate dehydrogenase	125-220 U/L	245	144	130	130
International normalized ratio	-	1.6	1.2	1.2	1.1
C-reactive protein	<5 mg/L	91.3	23.6	5.9	<1.0
Total bilirubin	0.20-1.20 mg/dL	0.73	0.42	0.43	0.91
Aspartate transaminase	5-34 U/L	27	17	36	23
Alanine transaminase	0-55 U/L	18	16	43	16
Alkaline phosphatase	40-150 U/L	47	50	74	61
Gamma-glutamyl transferase	<55 U/L	17	15	21	17
Creatinine	0.7-.3 mg/dL	0.9	0.6	0.7	0.8
Urea	19-44 mg/dL	28	20	24	37
Serum sodium	136-145 mmol/L	141	145	142	141
Serum potassium	3.5-5.1 mmol/L	4.1	3.9	4.2	4.9

**Figure 1:**
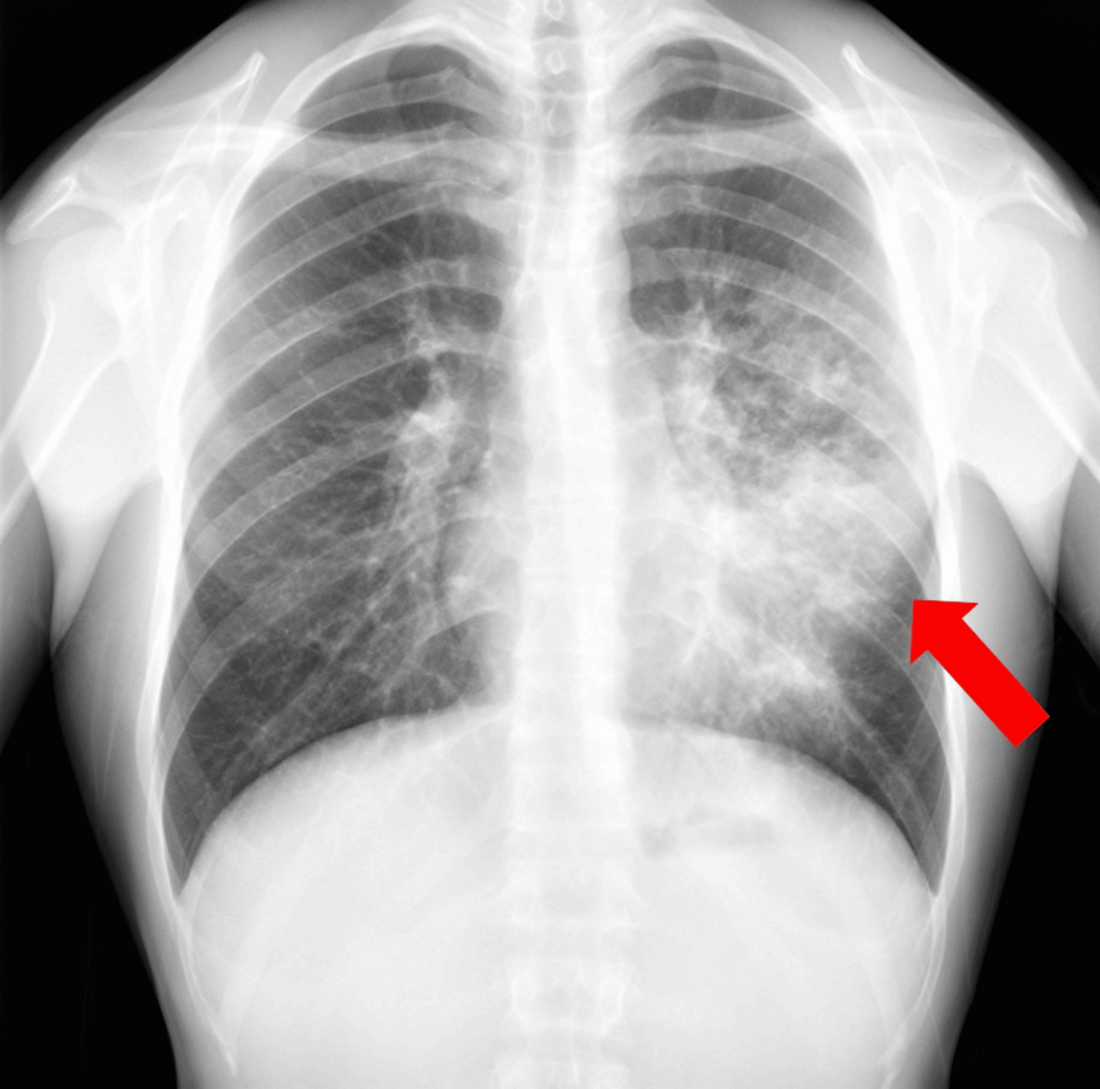
Chest radiography revealed extensive alveolar consolidation on the left hemithorax Arrow: extensive alveolar consolidation on the left hemithorax

**Figure 2:**
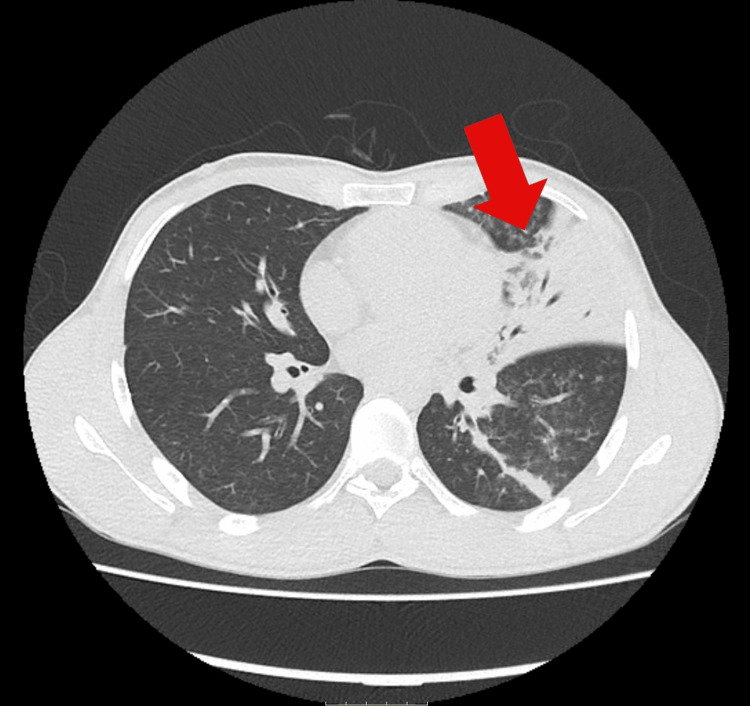
Chest CT revealing extensive parenchymal consolidation involving the left upper lobe, predominantly the lingular segment, and the posterior region of the ipsilateral lower lobe, accompanied by adjacent centrilobular opacities Arrow: extensive parenchymal consolidation involving the left upper lobe CT: computed tomography

Chest radiography revealed extensive consolidation on the left hemithorax (Figure 1), prompting a chest CT scan. CT demonstrated extensive parenchymal consolidation involving the left upper lobe, predominantly the lingular segment, and the posterior region of the ipsilateral lower lobe, accompanied by adjacent centrilobular opacities and a thin left pleural effusion (Figure 2).

The patient was admitted with a diagnosis of CAP of unknown etiology and started empirical antibiotic therapy with amoxicillin/clavulanate and azithromycin after collection of blood cultures (negative) and urinary antigen tests for *Streptococcus pneumoniae* and *Legionella pneumophila* (both negative).

Given the extent of pulmonary involvement, he was initially admitted to the intermediate care unit for monitoring. On the third day of hospitalization, new-onset anemia was noted (hemoglobin 12.9 g/dL, from an initial value of 14.1 g/dL), along with cold agglutinin-associated red blood cell agglutination that resolved after warming the specimen to 37°C, with suspected hemolysis; however, definitive confirmation was limited by the unavailability of a complete hemolysis evaluation during the acute phase, including haptoglobin, reticulocyte count, and direct antiglobulin testing. Additional evaluation (Table 2) revealed positive cold agglutinins and a positive Paul-Bunnell reaction, with negative Epstein-Barr virus serology (IgM and IgG) and positive *M. pneumoniae* serology (IgM and IgG). While serologic testing supported the diagnosis, its inherent limitations related to timing and potential false-positive results are acknowledged; respiratory PCR testing for *M. pneumoniae* was not available during the acute phase.

**Table 2:** Extended immunologic and microbiological workup Summary of immunologic, inflammatory, and microbiological investigations performed during hospitalization. Findings demonstrated elevated erythrocyte sedimentation rate, positive cryoglobulins, and serologic evidence of *Mycoplasma pneumoniae* infection (positive IgM and IgG), with all other infectious and autoimmune tests returning negative or within normal limits. ANA: antinuclear antibody, PCR: polymerase chain reaction, HIV: human immunodeficiency virus, IgM: immunoglobulin M, IgG: immunoglobulin G, EBNA: Epstein-Barr virus nuclear antigen, VCA: viral capsid antigen

Parameter	Reference range	Result
Erythrocyte sedimentation rate	0-12 mm	54
Immunoglobulin A	63-484 mg/dL	163
Immunoglobulin G	540-1822 mg/dL	1232
Immunoglobulin M	22-240 mg/dL	111
Rheumatoid factor	<30 UI/mL	<20
C3 complement	82-185 mg/dL	124
C4 complement	15-53 mg/dL	24
ANAs screening	-	Negative
Cryoglobulins	-	Positive
Blood cultures	-	Negative
Urinary *Legionella pneumophila* antigen	-	Negative
Urinary *Streptococcus pneumoniae* antigen	-	Negative
Respiratory virus PCR panel	-	Negative
HIV serology	-	Negative
Epstein-Barr virus	-	Epstein-Barr EBNA IgG negative; Epstein-Barr VCA IgG negative; Epstein-Barr VCA IgM negative
Hepatits B serology	-	Non-imunne
Hepatits C serology	-	Negative
Chlamydia pneumoniae	-	IgM negative; IgG positive
Mycoplasma pneumoniae	-	IgM positive; IgG positive

A diagnosis of *M. pneumoniae* pneumonia with extrapulmonary manifestations was established, and targeted therapy with azithromycin was continued for a total of seven days. Clinical and laboratory evolution was favorable, with resolution of anemia and a progressive decline in inflammatory markers, enabling discharge upon completion of antibiotic therapy. On follow‑up three months after discharge, the patient was asymptomatic, with no evidence of anemia, and erythrocyte agglutination was no longer present.

## Discussion

*M. pneumoniae* pneumonia usually presents as a mild to moderate respiratory illness; however, a broad spectrum of extrapulmonary manifestations may occur, including hematologic complications. These manifestations can arise at any point during the disease course, even in the absence of significant respiratory symptoms, and are predominantly mediated by immune mechanisms [4,7]. Among them, cold agglutinin-mediated hemolysis is an uncommon but well-recognized phenomenon. It results from the production of IgM antibodies directed against the erythrocyte membrane I antigen, leading to red blood cell agglutination at low temperatures and subsequent complement activation [5,6,8].

In this case, the development of new-onset anemia with erythrocyte agglutination after warming the blood sample was a key diagnostic clue, prompting early suspicion of immune-mediated hemolysis prior to serologic confirmation of *M. pneumoniae* infection.

The patient experienced a favorable clinical course with targeted antibiotic therapy and supportive measures, achieving complete resolution of hemolysis and normalization of inflammatory markers. This evolution aligns with previous reports indicating that *M. pneumoniae*-associated hemolysis is typically transient and self-limited once the underlying infection is treated [5,6,8]. Nonetheless, more severe presentations have been reported, including hemolytic anemia requiring transfusion and systemic complications [4,9]. These findings reinforce the need for close clinical and laboratory monitoring, particularly in the early stages of the disease.

Overall, this case highlights the importance of considering atypical pathogens in the evaluation of CAP in previously healthy young adults, especially when unexpected hematologic abnormalities are present. Early recognition of cold agglutinin-associated red blood cell agglutination with suspected hemolysis may accelerate diagnostic assessment and guide appropriate therapeutic intervention, thereby improving patient outcomes [1,3].

## Conclusions

This case illustrates that *M. pneumoniae* infections may extend beyond pulmonary involvement and include hematologic abnormalities that, although uncommon, can provide an important diagnostic clue in young adults presenting with CAP. In this patient, the presence of cold agglutinin-associated red blood cell agglutination, together with mild anemia, supported the suspicion of immune-mediated hematologic involvement, despite the absence of definitive objective markers of overt hemolysis. The favorable clinical course, with resolution of anemia and normalization of inflammatory parameters following targeted therapy, reinforces the typically self-limited nature of this complication when the underlying infection is promptly recognized and treated.

This report underlines the importance of maintaining a high index of suspicion when unexpected hematologic abnormalities arise, as these may precede or accompany respiratory symptoms. Careful integration of clinical, radiologic, and immunologic findings, along with prompt treatment and appropriate monitoring, leads to full recovery, as reported in prior case reports.
